# Supplementary material for: The coronavirus disease 2019 (COVID-19) vaccination psychological antecedent assessment using the Arabic 5c validated tool: An online survey in 13 Arab countries
Source: PLoS One. 2021 Nov 29;16(11):e0260321. doi: 10.1371/journal.pone.0260321 (PMC8629271; doi:10.1371/journal.pone.0260321)
Supplement: S1 Table — (DOCX) [file pone.0260321.s001.docx]

**S1 Table: Multivariate analysis of predictors affecting the 5C psychological antecedents**

| **Independent variables** | **Unstandardized Coefficients** | | **Odd Ratio** | **Sig.** | **95% C.I. for Odd Ratio** | |
| --- | --- | --- | --- | --- | --- | --- |
|  | **B** | **S.E.** |  |  |  |  |
|  |  |  |  |  | **Lower** | **Upper** |
| **Confidence** | | | | | | |
| **Constant** | -1.129 | 0.208 | 0.324 | <0.001* |  |  |
| **Female ^a^** | -0.356 | 0.086 | 0.701 | <0.001* | 0.592 | 0.829 |
| **Age** | 0.029 | 0.005 | 1.029 | <0.001* | 1.019 | 1.040 |
| **Education ^b^** |  |  |  | 0.024* |  |  |
| Technical/ vocational education | -0.280 | 0.285 | 0.756 | 0.325 | 0.433 | 1.320 |
| University degree | -0.345 | 0.159 | 0.708 | 0.030* | 0.519 | 0.966 |
| Postgraduate degree | -0.496 | 0.174 | 0.609 | 0.004* | 0.433 | 0.856 |
| Others | -0.689 | 0.238 | 0.502 | 0.004* | 0.315 | 0.800 |
| **Marital status ^c^** |  |  |  | 0.208 |  |  |
| Married | -0.216 | 0.110 | 0.805 | 0.050 | 0.649 | 1.000 |
| Divorced | -0.043 | 0.240 | 0.958 | 0.859 | 0.599 | 1.534 |
| Widowed | 0.310 | 0.359 | 1.031 | 0.932 | 0.510 | 2.085 |
| **Chronic status ^d^** | -0.089 | 0.120 | 0.915 | 0.460 | 0.724 | 1.157 |
| **HCP ^e^** | 0.067 | 0.091 | 1.069 | 0.462 | 0.894 | 1.278 |
| **Get COVID ^f^** | -0.588 | 0.108 | 0.555 | <0.001* | 0.450 | 0.686 |
| **COVID relative ^g^** | 0.234 | 0.091 | 1.264 | 0.01* | 1.057 | 1.511 |
| **Relatives died ^h^** | -0.219 | 0.093 | 0.803 | 0.019* | 0.669 | 0.964 |
| **Complacency** | | | | | | |
| **Constant** | -1.937 | 0.290 | 0.144 | <0.001* |  |  |
| **Female ^a^** | -0.075 | 0.124 | 0.927 | 0.545 | 0.727 | 1.184 |
| **Age** | 0.011 | 0.008 | 1.011 | 0.160 | 0.996 | 1.026 |
| **Education ^b^** |  |  |  | 0.045* |  |  |
| Technical/ vocational education | -0.349 | 0.395 | .705 | 0.377 | 0.325 | 1.531 |
| University degree | -0.314 | 0.211 | .730 | 0.138 | 0.483 | 1.106 |
| Postgraduate degree | -0.700 | 0.243 | .496 | 0.004* | 0.309 | .799 |
| Others | -0.312 | 0.304 | .732 | 0.306 | 0.403 | 1.329 |
| **Marital status ^c^** |  |  |  | 0.153 |  |  |
| Married | 0.234 | 0.162 | 1.264 | 0.148 | 0.920 | 1.736 |
| Divorced | 0.126 | 0.351 | 1.134 | 0.720 | 0.570 | 2.258 |
| Widowed | -1.587 | 1.036 | 0.205 | 0.126 | 0.027 | 1.560 |
| **Chronic status ^d^** | -0.342 | 0.186 | 0.710 | 0.066 | 0.493 | 1.023 |
| **HCP ^e^** | -0.669 | 0.143 | 0.512 | <0.001* | 0.387 | 0.678 |
| **Get COVID ^f^** | 0.442 | 0.145 | 1.556 | 0.002* | 1.171 | 2.068 |
| **COVID relative ^g^** | -0.249 | 0.135 | 0.780 | 0.065 | 0.599 | 1.016 |
| **Constraints** | | | | | | |
| **Constant** | -3.456 | 0.532 | .03 | <0.001* |  |  |
| **Male ^a^** | 0.188 | 0.215 | 1.207 | 0.383 | 0.791 | 1.841 |
| **Age** | -0.002 | 0.014 | 0.998 | 0.859 | 0.970 | 1.025 |
| **Education ^b^** |  |  |  | 0.165 |  |  |
| Technical/ vocational education | -0.024 | 0.702 | 0.976 | 0.972 | 0.246 | 3.866 |
| University degree | -0.115 | 0.392 | 0.891 | 0.770 | 0.413 | 1.923 |
| Postgraduate degree | -0.290 | 0.446 | 0.748 | 0.516 | 0.312 | 1.793 |
| Others | 0.629 | 0.479 | 1.877 | 0.189 | 0.734 | 4.798 |
| **Marital status ^c^** |  |  |  | 0.990 |  |  |
| Married | 0.092 | 0.277 | 1.096 | 0.740 | 0.637 | 1.886 |
| Divorced | 0.041 | 0.567 | 1.042 | 0.942 | 0.343 | 3.165 |
| Widowed | -17.923 | 6099.90 | 0.000 | 0.998 | 0.000 | . |
| **Chronic status ^d^** | -0.180 | 0.313 | 0.835 | 0.566 | 0.452 | 1.543 |
| **HCP ^e^** | -0.657 | 0.247 | 0.518 | 0.008* | 0.319 | 0.841 |
| **Get COVID ^f^** | 0.837 | 0.234 | 2.309 | <0.001* | 1.461 | 3.649 |
| **COVID relative ^g^** | -0.065 | 0.230 | 0.937 | 0.779 | 0.597 | 1.472 |
| **Relatives died ^h^** | 0.232 | 0.217 | 1.261 | 0.284 | 0.825 | 1.929 |
| **Calculation** | | | | | | |
| **Constant** | -1.055 | 0.196 | 0.348 | <0.001* |  |  |
| **Male ^a^** | 0.309 | 0.078 | 1.362 | <0.001* | 1.169 | 1.586 |
| **Age** | 0.012 | 0.005 | 1.012 | 0.014* | 1.002 | 1.021 |
| **Education ^b^** |  |  |  | <0.001* |  |  |
| Technical/ vocational education | -0.361 | 0.275 | 0.697 | 0.188 | 0.407 | 1.193 |
| University degree | 0.155 | 0.150 | 1.167 | 0.302 | 0.870 | 1.565 |
| Postgraduate degree | 0.378 | 0.162 | 1.459 | 0.02* | 1.061 | 2.007 |
| Others | -0.279 | 0.215 | 0.757 | 0.194 | 0.497 | 1.153 |
| **Marital status ^c^** |  |  |  | 0.712 |  |  |
| Married | 0.056 | 0.098 | 1.057 | 0.569 | 0.873 | 1.282 |
| Divorced | -0.049 | 0.219 | 0.952 | 0.824 | 0.621 | 1.462 |
| Widowed | 0.356 | 0.344 | 1.428 | 0.301 | 0.728 | 2.801 |
| **Chronic status ^d^** | -0.128 | 0.108 | 0.880 | 0.235 | 0.712 | 1.087 |
| **HCP ^e^** | 0.237 | 0.081 | 1.268 | 0.003* | 1.082 | 1.486 |
| **Get COVID ^f^** | -0.063 | 0.091 | 0.939 | 0.491 | 0.786 | 1.123 |
| **COVID relative ^g^** | 0.009 | 0.082 | 1.010 | 0.908 | 0.860 | 1.185 |
| **Relatives died ^h^** | 0.221 | 0.081 | 1.248 | 0.007* | 1.064 | 1.463 |
| **Collective responsibility** | | | | | | |
| **Constant** | -0.824 | 0.197 | 0.439 | <0.001* |  |  |
| **Male ^a^** | -0.102 | 0.078 | 0.903 | 0.192 | 0.774 | 1.053 |
| **Age** | 0.014 | 0.005 | 1.014 | 0.005* | 1.004 | 1.023 |
| **Education ^b^** |  |  |  | 0.3 |  |  |
| Technical/ vocational education | -0.028 | 0.270 | 0.972 | 0.917 | 0.573 | 1.649 |
| University degree | 0.090 | 0.151 | 1.094 | 0.550 | 0.814 | 1.472 |
| Postgraduate degree | 0.041 | 0.164 | 1.041 | 0.805 | 0.755 | 1.437 |
| Others | -0.288 | 0.219 | 0.750 | 0.188 | 0.488 | 1.152 |
| **Marital status ^c^** |  |  |  | 0.219 |  |  |
| Married | -0.148 | 0.099 | 0.862 | 0.136 | 0.710 | 1.048 |
| Divorced | -0.427 | 0.228 | 0.652 | 0.061 | 0.417 | 1.021 |
| Widowed | -0.164 | 0.348 | 0.848 | 0.637 | 0.429 | 1.678 |
| **Chronic status ^d^** | -0.059 | 0.109 | 0.942 | 0.587 | 0.761 | 1.167 |
| **HCP ^e^** | 0.466 | 0.082 | 1.594 | <0.001* | 1.358 | 1.872 |
| **Get COVID ^f^** | -0.489 | 0.094 | 0.613 | <0.001* | 0.510 | 0.736 |
| **COVID relative ^g^** | 0.232 | 0.083 | 1.261 | 0.005* | 1.072 | 1.482 |
| **Relatives died ^h^** | -0.046 | 0.083 | 0.955 | 0.58 | 0.812 | 1.123 |

*; Statistically sig; ^a^ ref; Male; ^b^ ref; pre-university; ^c^ ref; single; ^d^ ref; No chronic status; ^e^ ref; No HCP;

^f^ ref; Not getting COVID; ^g^ ref: relative not getting COVID; ^h^ ref; no relative died
